# Artificial intelligence-supported web application design and development for reducing polypharmacy side effects and supporting rational drug use in geriatric patients

**DOI:** 10.3389/fmed.2023.1029198

**Published:** 2023-03-08

**Authors:** Seyma Handan Akyon, Fatih Cagatay Akyon, Tarık Eren Yılmaz

**Affiliations:** ^1^Family Medicine Department, Ankara City Hospital, University of Health Sciences, Ankara, Türkiye; ^2^Graduate School of Informatics, Middle East Technical University, Ankara, Türkiye

**Keywords:** artificial intelligence, computer-assisted decision making, drug interactions, family practice, geriatrics, multimorbidity, polypharmacy, potentially inappropriate medication list

## Abstract

**Introduction:**

The main complications of polypharmacy, which is known as the simultaneous use of more than five drugs, are potentially inappropriate medicines(PIMs), drug–drug, and drug-disease interaction. It is aimed to prepare an auxiliary tool to reduce the complications of polypharmacy and to support rational drug use(RDU), by evaluating the patient with age, drugs, and chronic diseases in this study.

**Materials and methods:**

In the first phase of this study, as methodological research, an up-to-date and comprehensive auxiliary tool as a reference method was generated with a database containing interaction information of 430 most commonly used drug agents and chronic diseases in geriatrics in the light of current and valid 6 PIM criteria for geriatric patients, and medication prospectuses, relevant current articles, and guidelines. Then, an artificial intelligence(AI) supported web application was designed and developed to facilitate the practical use of the tool. Afterward, the data of a cross-sectional observational single-center study were used for the rate and time of PIM and drug interaction detection with the web application. The proposed web application is publicly available at https://fastrational.com/.

**Results:**

While the PIM coverage rate with the proposed tool was 75.3%, the PIM coverage rate of EU(7)-PIM, US-FORTA, TIME-to-STOPP, Beers 2019, STOPP, Priscus criteria in the web application database respectively(63.5%–19.5%) from the highest to the lowest. The proposed tool includes all PIMs, drug–drug, and drug-disease interaction information detected with other criteria. A general practitioner detects interactions for a patient without the web application in 2278 s on average, while the time with the web application is decreased to 33.8 s on average, and this situation is statistically significant.

**Discussion:**

In the literature and this study, the PIM criteria alone are insufficient to include actively used medicines and it shows heterogeneity. In addition, many studies showed that the biggest obstacle to drug regulation in practice is “time constraints.” The proposed comprehensive auxiliary tool analyzes age, drugs, and diseases specifically for the patient 60 times faster than the manual method, and it provides quick access to the relevant references, and ultimately supports RDU for the clinician, with the first and only AI-supported web application.

## Introduction

The term polypharmacy is generally known as the simultaneous use of five or more drugs. However, not only in terms of the number of medicines but also in inappropriate indications and the combined use of two or more unnecessary medicines is called polypharmacy (1–3). There are two risk factors leading to polypharmacy. These are patient-related and health system-related risk factors and are given in Table 1 (1, 4–6). When patient-related factors are examined; the number of medicines used for the chronic disease increases with age, so polypharmacy increases with age (7). However, the coexistence of many diseases (multimorbidity) in individuals regardless of age also leads to polypharmacy and an increase in related side effects (8). The main causes of polypharmacy originating from the health system are the insufficient number of primary care physicians to coordinate medicine treatment or the physician’s insufficient knowledge about the side effects of the medicine and drug–drug interactions (7). In addition, the “prescription cascade,” defined as the administration of another medicine to treat the side effect of a given medicine, is another important cause of polypharmacy (9, 10). The complications of polypharmacy are listed in Table 1 and the main ones are adverse drug events (ADEs), potentially inappropriate medicine (PIM) use in the elderly, drug–drug interaction, drug-disease interaction, and increased treatment costs, increased hospitalization, and increased mortality (1, 4, 7, 11, 12). Drug side effects increase with the number of medicines used (9).

**Table 1:** Risk factors and side effects of polypharmacy.

Risk factors of polypharmacy	Complications of polypharmacy
**Patient-related**Being over the age of 62Multiple chronic diseasesMultiple symptomsCognitive ImpairmentDevelopmental impairmentFragilityMental health conditionsSelf-treatmentResidency in a long-term care facility**Health system related**Keeping a poor medical recordInsufficient number of primary care physicians to coordinate medicine treatmentMultiple prescriptionsPrescription cascadeInsufficient knowledge of the physician about medicine side effects and drug–drug interaction	Potentially inappropriate medicine useIncreased risk of adverse drug eventsDrug–drug interactionDrug-disease interactionIncreased burden on the health systemDecrease in doctor functionality and quality of careIncrease in costFall and/or hip fractureCognitive dysfunction and sedationIncrease in nursing home placementFragilityDeath

PIMs, on the other hand, refer to the use of any medicine with a high risk in terms of off-indication or side effects, and it increases in elderly patients and the presence of polypharmacy, and it also increases the risk of ADEs. Therefore, it is important to determine the use of PIM in elderly patients who have an increased risk of polypharmacy and who are more sensitive to ADEs and to organize treatment in the light of rational drug use principles (13). Because ADE, as one of the side effects of polypharmacy, can also be minimized by reducing the number of drugs and especially inappropriate drug use. To identify and prevent PIM use in the elderly, criteria were published around the world in the last three decades (9, 14). One of the common guidelines used for PIMs in the elderly is the Beers criteria (9). The Beers criteria were first published in 1991, and last updated in 2019 (15). As the Beers criteria were not sufficient in practical use, new criteria named STOPP (Screening Tool of Older Person’s Prescriptions) and START (Screening Tool to Alert Doctors to the Right Treatment) were created in Ireland in 2008 and they were updated in 2018 (9, 16, 17). In Turkey, it was aimed to create criteria for PIM in the elderly specific to their own society, and TIME (Turkish Inappropriate Medication Use in the Elderly) criteria were established (18). In addition to these criteria FORTA (Fit For The Aged), PRISCUS, EU (7)-PIM lists are among PIM use criteria accepted in various European countries (19–24). However, there is a need for newer, more up-to-date, more comprehensive, system-based, and easily applicable criteria and tools that can be applied in routine clinical practice due to the deficiency and insufficiency of each of the criteria identifying PIM (25).

Many factors contribute to the relevance and quality of prescribing medicines. These include deciding that a medicine is indicated, choosing the best medicine, proper use of selected medicines, avoiding PIMs, monitoring ADEs and medicine levels, and avoiding drug–drug and drug-chronic disease interactions. Even if the clinician applies the clinical practice guidelines for the patient’s diseases, the guidelines prepared specifically for the disease may lead to polypharmacy side effects by ignoring the patient’s comorbidity and additional medications. That is why a more systematic approach is required to guide the tailoring of drug regimens according to the individuals’ needs (2). According to the principle of “first, do no harm,” which is one of the main ethical criteria of the medical profession, it is necessary to avoid iatrogenic damages due to over-screening, diagnosis, and over-treatment when applying preventive health services. This situation brought about the concept of quaternary protection that supports rational drug use (26, 27). The concept of “deprescribing” refers to the gradual discontinuation of PIMs, and it is supervised by a healthcare professional. It is an important part of quaternary prevention and its purpose is to ensure the safe and effective use of appropriate medicines and to protect patients from inappropriate overuse, and the side effects of polypharmacy. This process requires time, awareness, and special skills and knowledge (28, 29). In addition, family physicians are expected to coordinate and advocate for patients involved in primary care management, which is one of the core competencies of the family medicine discipline, and due to a comprehensive, person-centered, longitudinal, and holistic approach, family physicians are expected to be more careful in these matters and to manage the control of the drugs prescribed by physicians from other branches more carefully in their own patient’s situation (30).

Recently, the increase in the elder population and chronic diseases due to epidemics such as the COVID-19 pandemic, disasters, and prolonged life expectancy caused an increase in the workload and a decrease in the time of a clinician to care for the patients (31). For such reasons, the place and importance of web applications and artificial intelligence (AI) are increasing in the health field and in our lives (32). AI-aided applications are used to diagnose diseases, predict prognosis after treatment, and make decisions to help the clinician in healthcare services (32). Currently, the main AI methods are expert systems (ESs), fuzzy logic, artificial neural networks (ANNs), and genetic algorithms (33). Expert systems consist of a database containing data, rules, relationships, problem definitions, solutions, and information about the solution, as well as an inference mechanism providing analysis of these data and rules (34). Expert systems aim to provide information in a cause-and-effect relationship with patient data, and expert systems in the field of medicine are not intended to replace the doctor, but to make recommendations to the doctor based on patient data (33, 35). Rule-based systems, causal models, and hypothesis-based systems are some examples of expert systems (33).

Currently, there are some web applications for drug–drug interaction detection such as UpToDate, Lexicomp, Vademecum online, Medscape online drug interaction, Webmd drug interaction, and DDInter (36–42). Also, there are some online sites created with certain algorithms that will support and facilitate deprescribing elderly patients in the clinician’s practice (43–47). Although they are important tools for rational drug use, they provide more standard information rather than a patient-centered approach because they ignore the existing diseases, medications, or age of the patient. Therefore, in order to assist healthcare professionals in their daily practices, it was aimed to prepare an auxiliary tool that will evaluate the patient with their age, medications, and chronic diseases in the light of current and valid medical criteria, medication prospectuses, relevant articles, and guidelines. Then, to use this tool easily and quickly in practice it was aimed to design an English language and local language-supported web database containing the frequently prescribed pharmaceutical agents and common comorbid diseases in elderly patients. In the web application to be prepared, the pharmaceutical agents and chronic diseases of a geriatric patient are evaluated together and drug–drug interactions, drug-chronic disease interactions, and PIMs for over 65 years of age are analyzed at the same time, and it is aimed to provide a great convenience for the rational drug use in the clinician’s practice.

## Materials and methods

This research is a cross-sectional, observational, single-center study. An artificial intelligence-supported web application was designed and developed as a result of methodological research based on the data of a single-center study.

**Figure 1:**
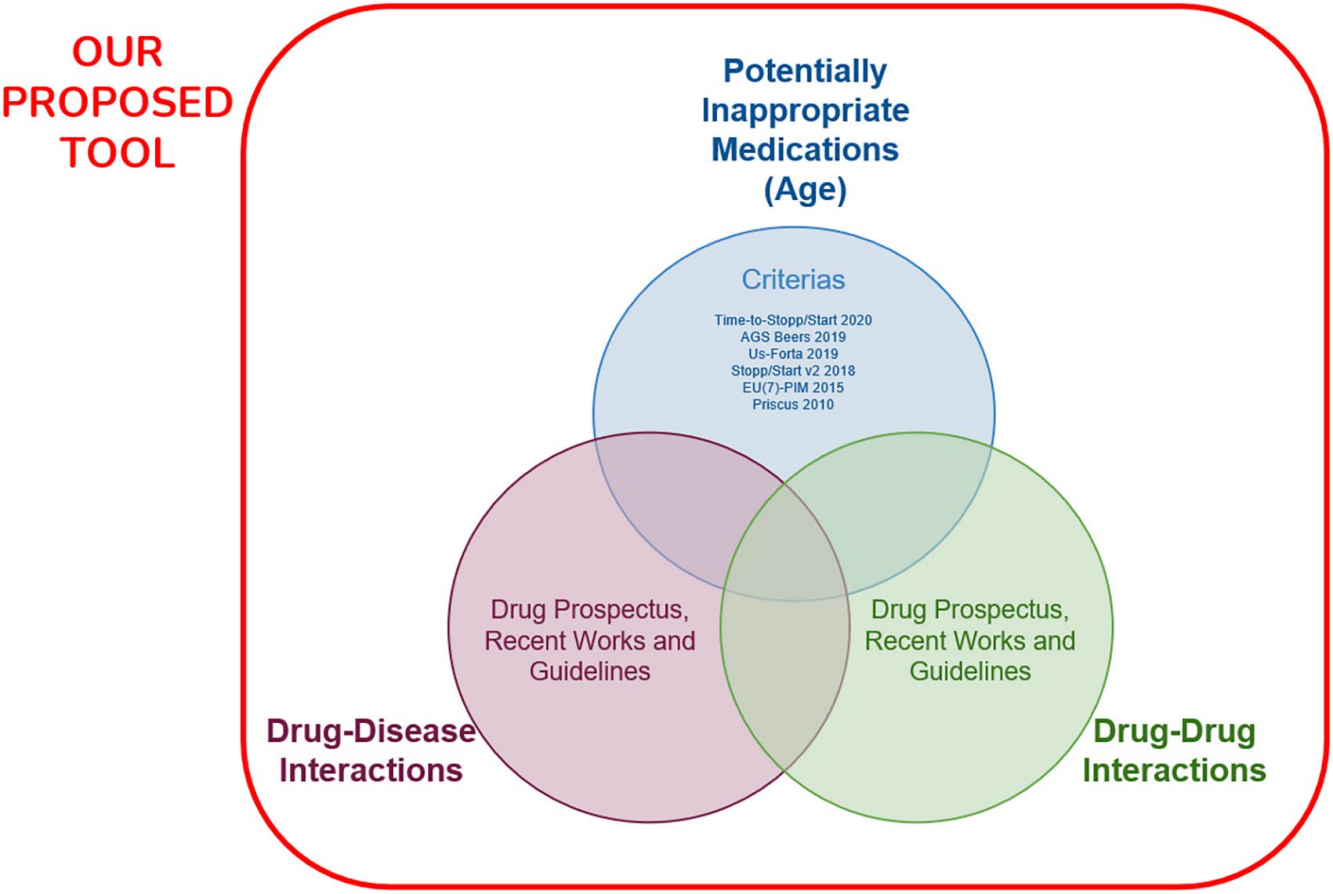
The proposed comprehensive compiler utility tool as the reference method.

Firstly, a database was created in light of current and valid PIM criteria for geriatric patients, medication prospectuses, current peer-reviewed articles published on drug–drug interaction and drug-disease interaction, and guidelines to build a comprehensive compiler utility tool (Figure 1). In order to create the database and algorithms of the auxiliary tool, frequently used pharmaceutical agents in daily practice and chronic diseases and clinical conditions frequently encountered in geriatric patients were identified. For this purpose, 1,203 active pharmaceutical agents licensed from the Turkey Pharmaceuticals and Medical Devices Agency (TITCK) 2022 E-prescription Drug List were identified (48). Each pharmaceutical agent was screened by the researcher and the ones that are not available in the market, with their licenses revoked, in the overseas drug category and parenteral forms of them were eliminated, and the pharmaceutical agents were simplified to the enteral forms on the market. Among the simplified pharmaceutical agents, 430 most frequently used pharmaceutical agents were identified in the light of the 2016 report, which included the top 100 drugs with the highest sales value of the TITCK (49–51). The pharmaceutical agents detected later were classified according to the medical classification system ATC (Anatomical Therapeutic Chemical Classification) which separates them by their effective organs or systems and their structural, therapeutic, pharmacological, chemical, and chemical compound properties. Also, ATC is supported, managed, and developed by the World Health Organization (52). The most common 73 chronic diseases and medical conditions in elderly patients were compiled to be used in the web application database, which will be in addition to frequently used drug agents.

Three separate tables which are drug-age, drug–drug, and drug-disease interaction were created in the database. When creating the first table of the database, the aforementioned 430 drug agents were screened with 6 PIM criteria [Beers 2019 Criteria, Time-to-STOP/START Criteria, STOPP/START Criteria, Priscus Criteria, US-FORTA Criteria and EU (7)-PIM List] and classified in three different categories as “usable,” “risky,” and “no warning was found” in patients over 65 years of age. When creating the second table of the database, interaction information of these 430 drug agents was gathered from drug prospectuses, pharmacology medical books, and related articles published in peer-reviewed journals. According to these sources, interaction information was classified into 3 different categories “risky,” “contraindicated,” and “no warning was found.” When creating the third table of the database, drug-disease interaction information was gathered from drug prospectuses, chronic disease guides, pharmacology medicine books, and articles published in peer-reviewed journals. According to these sources, interaction information was classified into three different categories “risky,” “contraindicated,” and “no warning was found.” Additional information about the interaction in three categories was added to each table along with the sources from which it was taken as comments.

**Figure 2:**
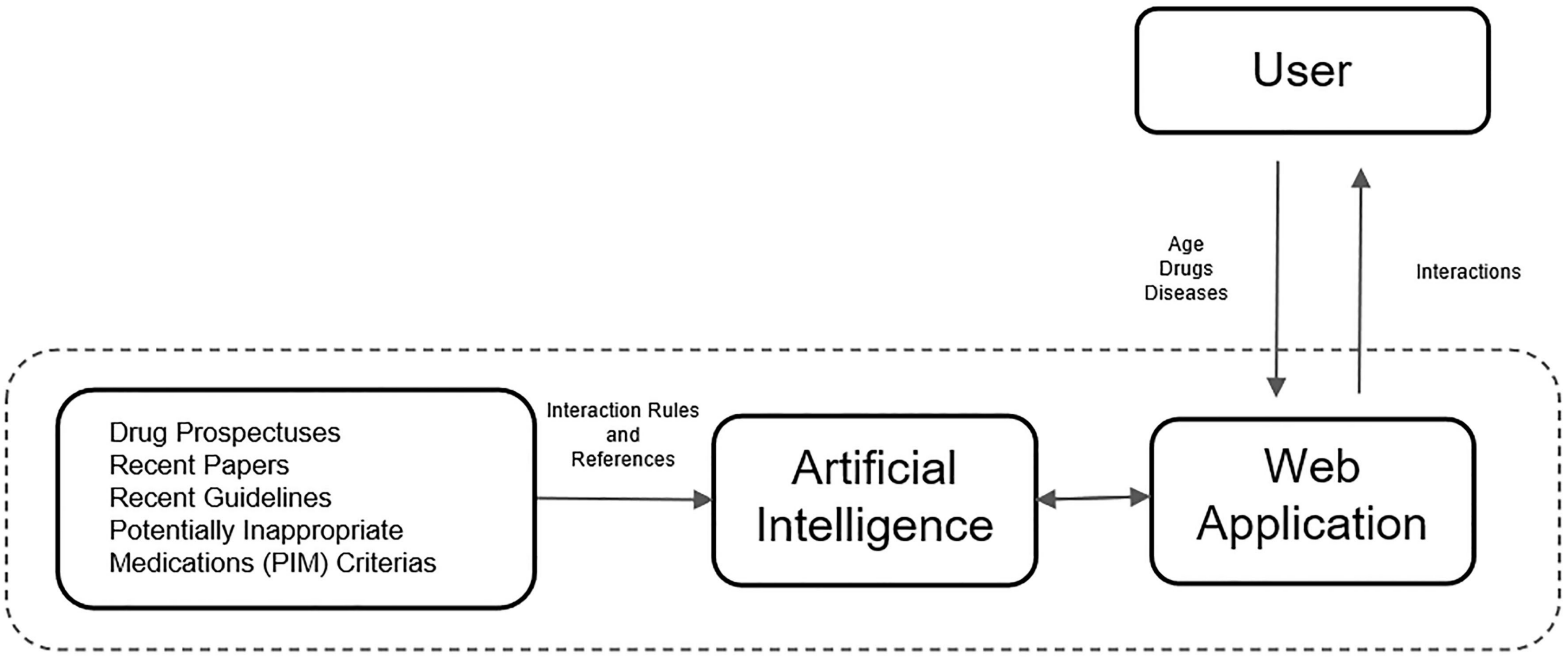
The flow chart illustrates the flow from the proposed tool to the proposed web application.

**Figure 3:**
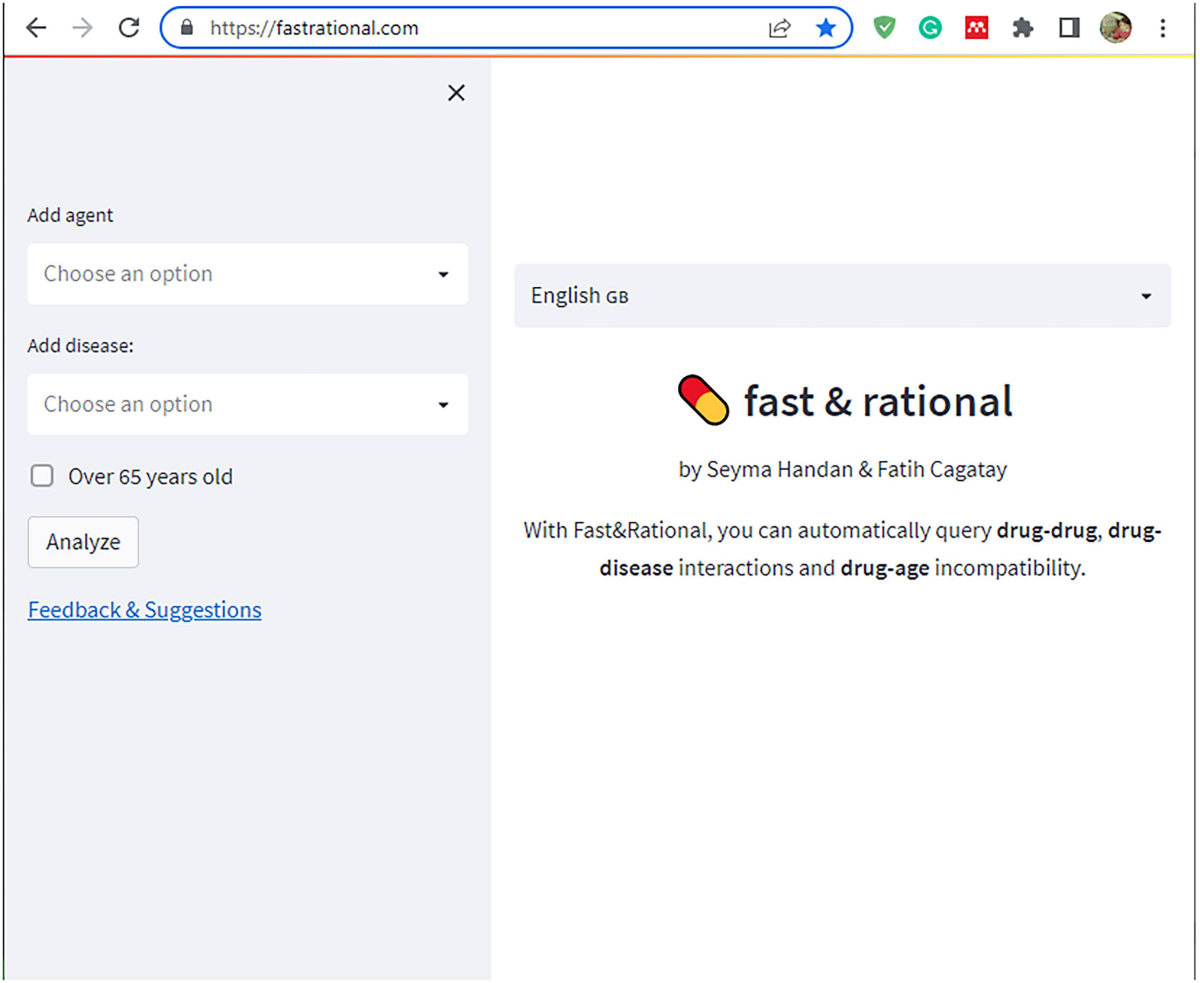
The interface of the proposed web application’s entrance.

**Figure 4:**
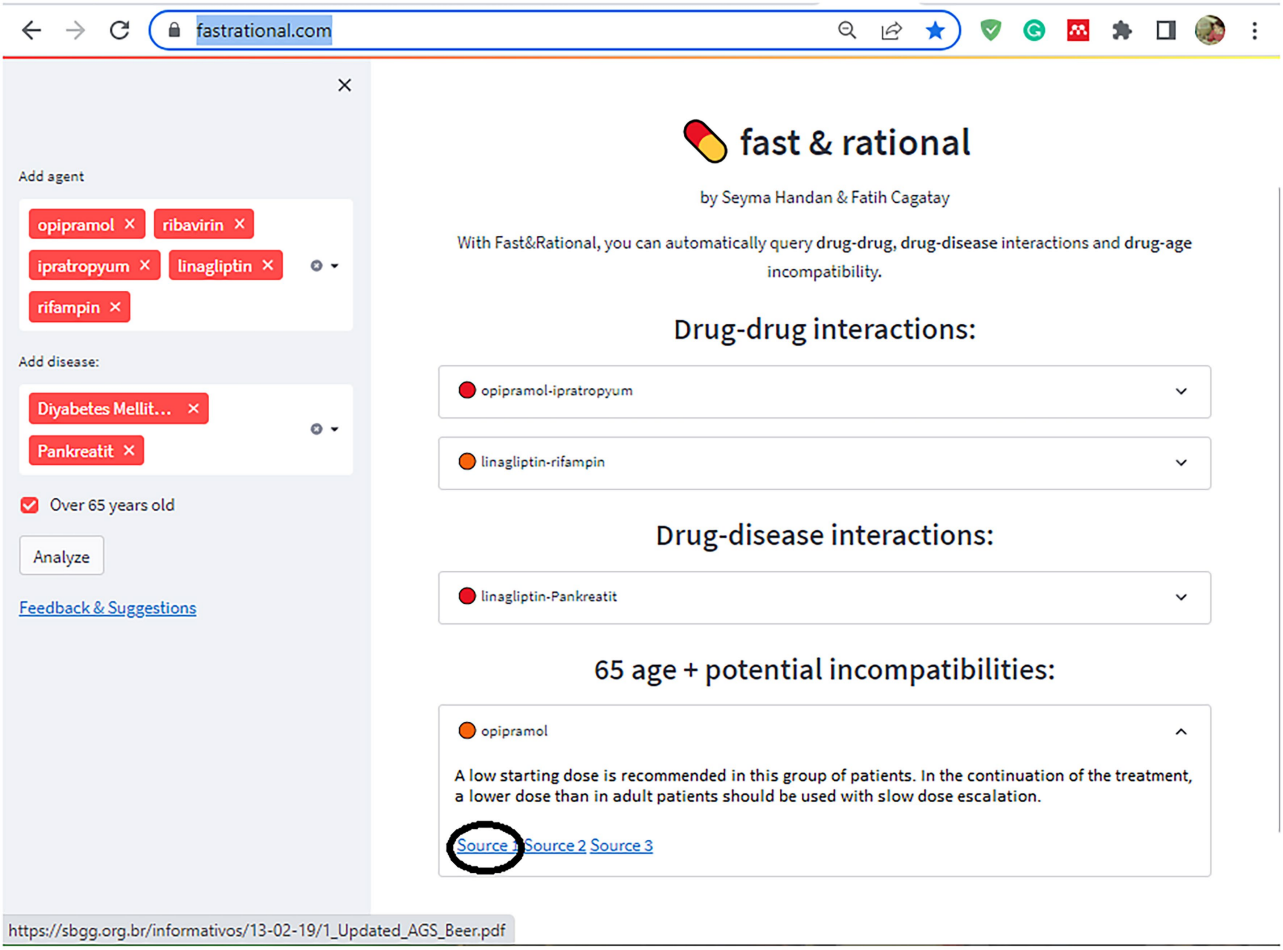
The interface of the proposed web application shows input, output, and resources as an example.

**Figure 5:**
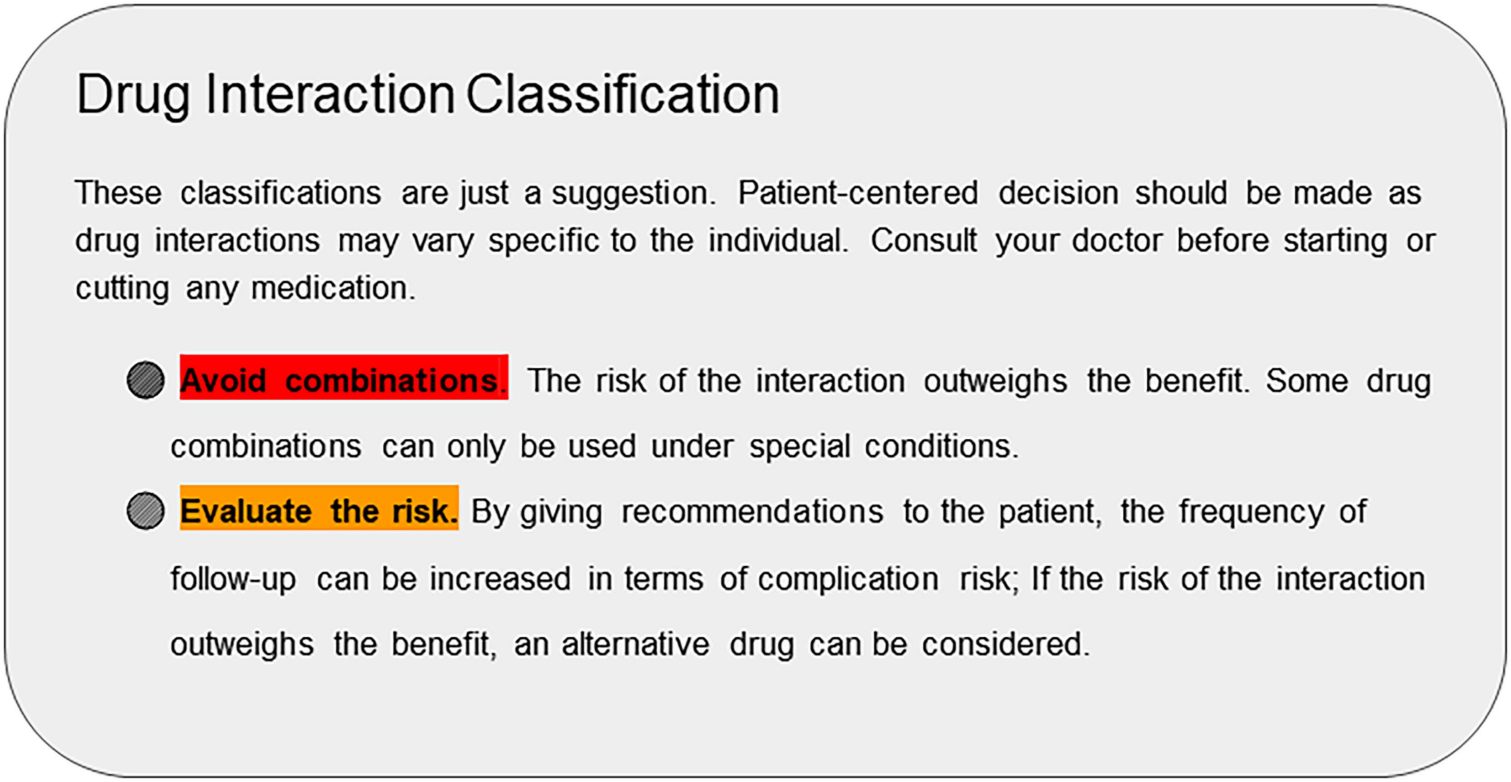
The interaction classification of the web application’s output.

In the second stage of this study, a rule-based artificial intelligence-supported web application was designed so that the tool, which will evaluate the patient with age, medications, and chronic diseases, can be used quickly and easily in daily practice. A rule-based artificial intelligence-based algorithm works on top of this database and is able to retrieve the queried interaction information in under a second. Since our proposed AI system uses predefined rules extracted from various drug prospectuses, pharmacology medical books, and related articles published in peer-reviewed journals, its decisions are determinant and explainable. The system also retrieves the related source for the decision for further justification. Lastly, the front-end of our web application is developed with Streamlit which is a Python and Javascript-based framework. The flow chart of this study is illustrated in Figure 2. The rule-based AI-aided web application’s interface design was developed with the Python programming language using the Streamlit Library. The web application’s interfaces with entrance, input, output, and sources are shown in Figures 3, 4. The interaction classification of the web application’s output is shown in Figure 5.

The articles and criteria published in peer-reviewed journals that we used in the database of the web application we suggested were accepted as the gold standard. PIMs were detected in an earlier study by manually analyzing the sample data with the Beers 2012 Criteria and the second version of the STOPP/START Criteria (51). In this study, the scope of criteria and resources to be used in the database on inappropriate drug use in the elderly were expanded to Beers 2019 Criteria, Time-to-STOP/START Criteria, STOPP/START Criteria, Priscus Criteria, US-FORTA Criteria, and EU (7)-PIM List. As a reference method, an auxiliary tool was created covering the entire content of these six criteria. PIM detection rates of this reference method with the screening result of six criteria in the database together, the PIM detection rates of each criterion, and the results of previous studies were compared. In addition to the sample data, 430 pharmaceutical agents used in the auxiliary tool database were screened separately for PIM according to each PIM criterion and compared with the PIM detection rates of our reference method, which evaluates 6 criteria together.

Detection rates for drug–drug and drug-disease interactions in sample data of reference method which includes medication prospectuses containing interaction information, 6 PIM criteria together, and the current articles compiled from the PUBMED database were compared with detection rates for drug–drug and drug-disease interactions of Time-to-STOPP, Beers 2019, and STOPP v2 (version 2) Criteria.

After the web application design was built, in the last stage of this study, the age, medications, and chronic diseases information of 296 geriatric patients registered in the Ankara Numune Training and Research Hospital Home Care Unit, one of the largest tertiary care hospitals in the capital city Ankara between 2011 and 2018 were used. The above-mentioned resources were Google-scanned manually and the total time taken to reach the information on PIMs, drug–drug interactions, and drug-disease interaction information for each patient over 65 years of age was noted in seconds. Then, with our web application, which was designed with the same patient information, all three categories were evaluated together and the total printout time was calculated. Finally, this printout time was compared with the printout time as a result of the literature research with the manual method.

Descriptive statistics were made with the IBM SPSS Statistics 28 program and the statistical significance level was accepted as *p* < 0.05. In descriptive statistics, numerical data are given as mean and standard deviation, and categorical data are given as numbers and percentages. Conformity of continuous variables to normal distribution was examined with the Skewness–Kurtosis test. Pearson and Spearman were used in the analysis of continuous variables, and Chi-square tests were used in the analysis of categorical variables. On the same data, whether the mean difference was significant in terms of duration and detection of PIM use before and after the web application was tested through the dependent sample *t*-test.

Ethics committee approval (Document Date: 08/12/21, Document Number: E.Kurul-E2-21-1083) was taken from the local research ethics committee for the research.

## Results

There are a total of 430 pharmaceutical agents in the database of the proposed tool and web application in this study. These pharmaceutical agents were screened with the tool covering 6 PIM criteria (AGS Beers 2019, TIME-to-STOPP, STOPP v2, EU (7)-PIM, US-FORTA, Priscus). To what extent the 6 PIM Criteria include the 430 agents was calculated. Accordingly, out of 430 agents, the criteria containing the most information about the pharmaceutical agents are US-FORTA (54.1%), Time-to-STOPP (49.5%), and EU(7)-PIM (41.6%), respectively. Among the 430 agents, Time-to-STOPP Criteria cover 97.4% of STOPP v2 criteria, while STOPP Criteria cover 53.1% of Time-to-STOPP criteria. Therefore, it was determined that the Time-to-STOPP Criteria are more comprehensive than the STOPP v2 criteria. Among the pharmaceutical agents included in the proposed tool, 5 out of 6 criteria (US-FORTA, Time-to-STOPP, AGS Beers 2019, Priscus, EU(7)-PIM criteria) in the proposed tool database, there were drug agents evaluated as PIM at least by 3 of them. It was calculated proportionally how much of the 78 drug agents determined as PIM were determined by the five criteria. Also, EU(7)-PIM (86%) determined the highest rate of PIMs. Time-to-STOPP (93.3%), AGS Beers 2019 (77%), and US-FORTA (76%) followed this criterion. According to these results, it was determined that the EU(7)-PIM list drug coverage and PIM detection rates were higher than the other criteria. According to the proposed tool, 201 (46.7%) of the relevant drug agents were found to be inappropriate in geriatric patients, 139 (32.3%) were found to be usable in geriatric patients, and no warning was found in any of the criteria for 90 (20.9%) pharmaceutical agents.

According to the patient data included in our sample, 25.7% (*n* = 76) of the total 296 geriatric patients were male and 74.3% (*n* = 220) were female. The mean age of the patients was 65–103 years and the mean age was determined as 83 ± 8 in men and 86 ± 7 in women. A patient has an average of 3.62 ± 1.40 chronic diseases in total and uses a total average of 5.33 ± 2.9 drugs (minimum 0, maximum 14). In men, this rate is 3.5 ± 1.5 chronic diseases on average, and they use 6.1 ± 3.1 medicines on average. On the other hand, women have an average of 4.0 ± 1.4 chronic diseases and use an average of 5.3 ± 2.9 medications. While 64.9% (*n* = 192) of the patients were taking at least 5 or more medications, 8.8% (*n* = 26) were taking at least 10 or more medications. According to patient data, each patient has at least 1 chronic disease, and the rate of multimorbidity (having two or more chronic diseases) is 97.3% (*n* = 288). Moreover, about half of the patients have four or more chronic diseases. The most common chronic disease is hypertension (69.5%), followed by chronic kidney disease (42.2%) and dementia (33.1%).

There are 201 different pharmaceutical agents among the medicines used by 296 patients. Among the 201 different pharmaceutical agents used by the patients, the 5 most commonly used pharmaceutical agents were acetylsalicylic acid with 52.3% (*n* = 99), proton pump inhibitor with 46.5% (*n* = 88), hydrochlorothiazide with 40.7% (*n* = 77), metoprolol with 30.6% (*n* = 58), and quetiapine with 26.9% (*n* = 51), respectively. In the proposed web application tool, it was observed that this sample included 189 (94%) of the 201 pharmaceutical agents.

**Figure 6:**
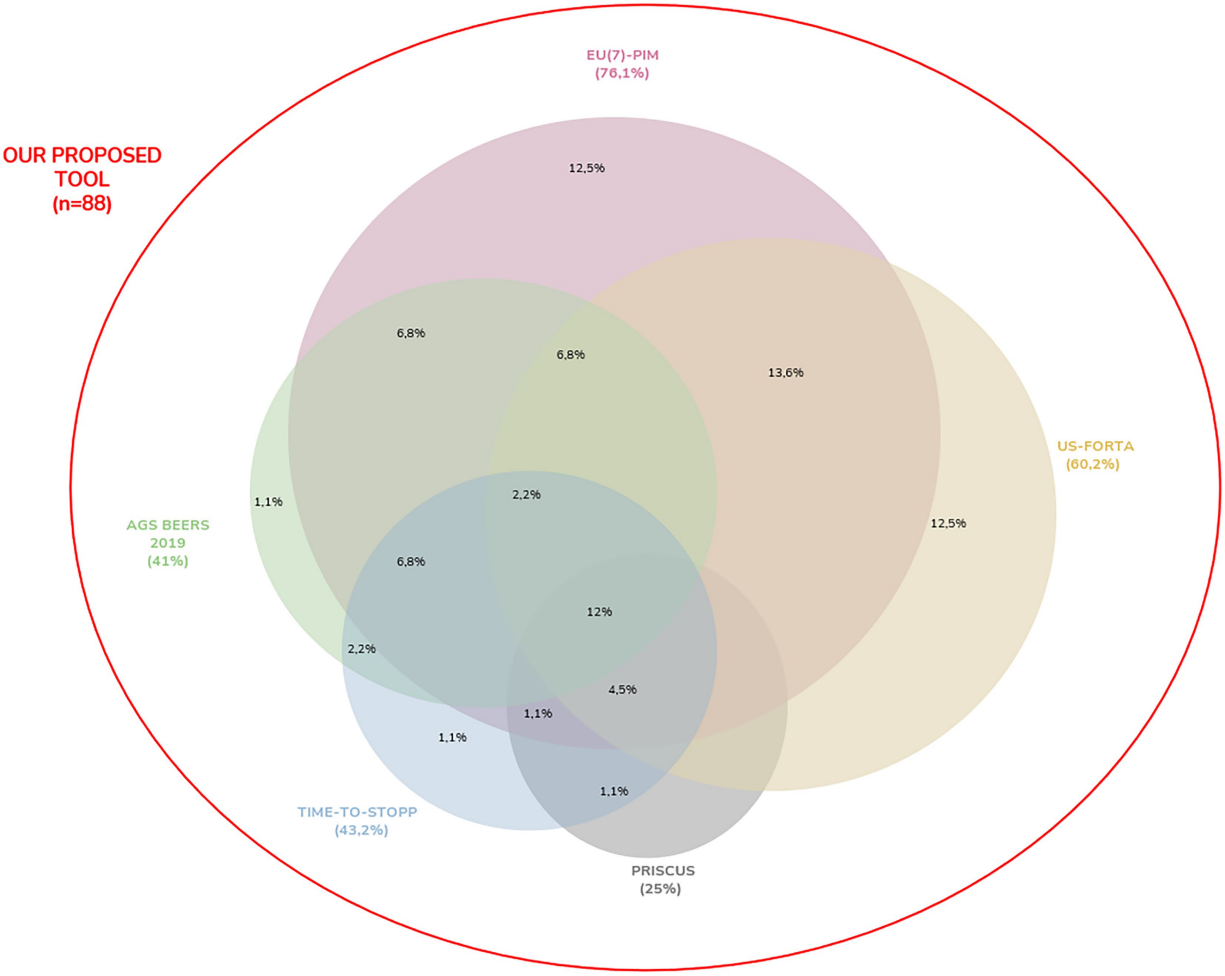
The Venn diagram illustrates the overlap between the six different PIM criteria and the proposed tool which includes all six PIM criteria among 88 PIM detected by the reference method. Of the included PIMs, 12% were identified within all six criteria.

All drug interaction information obtained from the bibliography, which is accepted as the gold standard, as a result of the statistical analyzes performed on the data of 296 patients, outputs of the proposed web application, matched exactly with the drug interaction information in all three categories obtained with the web application we proposed. When patient data were screened for potential inappropriate drug use in the elderly with the reference method (regardless of the disease and other medicines used), 88 (46.5%) drugs were found to be PIM, 90 (47.6%) “usable” and 11 pharmaceutical agents were determined as “no warning found.” 88 pharmaceutical agents detected as PIM with the reference method including 6 criteria together were screened separately with other PIM criteria, and the rate of PIM they detected is shown on the Venn diagram in Figure 6. According to this diagram, EU(7)-PIM (76.1%), US-FORTA (60.2%), TIME-to-STOPP (43.2%), AGS beers 2019 (41%), and PRISCUS (25%) are the criteria that contain the highest rate of PIM regardless of the disease and other drugs used, respectively. The PIM coverage rate by the EU(7)-PIM and US-FORTA Criteria alone are the same, and this rate is 12%.

The average PIM rates per patient, the rate of patients with at least 1 or more PIM detected, and the average PIM values determined in these patients were compared independently from the medications they use and their chronic diseases according to the proposed tool as the reference method, each criterion which was included by reference method, and also AGS Beers 2012 criterion. These were given in Table 2 with comparisons. The PIM rate determined by the reference method is higher than the PIM coverage rates of the other six criteria separately, and this is statistically significant for each criterion (*p* < 0.001). According to the table, the detection rate of a patient with at least 1 PIM use is 75.3% in the patient data, the average number of PIM use per patient is 1.63 ± 1.46, and the mean PIM number is calculated as 2.16 ± 1.3 for each patient with at least 1 PIM use for the reference method evaluating 6 separate PIM criteria together. According to the reference method, while men have an average of 2 PIM per patient, regardless of the additional disease and other medications they use, women have an average of 1 per patient. Compared to other criteria, the criterion that contains the highest percentage of PIM with 63.5% is EU(7)-PIM. When AGS Beers 2019 and AGS Beers 2012 were compared, the amount of PIM contained by Beers 2019 was higher and was statistically significant (*p* < 0.001). In addition, when Time-to-STOPP and STOPP v2 Criteria were compared, TIME-to-STOPP Criteria were found to be superior in containing PIM (*p* < 0.001).

**Table 2:** Comparison of PIM coverage rates of the proposed tool as reference method and each criterion.

	Reference method	EU(7)-PIM	US-FORTA	TIME-TO-STOPP	AGS BEERS 2019	STOPP V2	AGS BEERS 2012	Priscus
Mean number of PIM in all patients	1.63 ± 1.46	1.12 ± 1.14	0.9 ± 0.99	0.83 ± 0.88	0.82 ± 0.91	0.67 ± 0.76	0.65 ± 0.77	0.24 ± 0.55
Rate of patients using at least 1 PIM use (%)	75.3%	63.5%	57.7%	57.4%	55.74%	51%	48.6%	19.5%
Mean Number of PIM in patients with PIM	2.16 ± 1.3	1.77 ± 0.95	1.57 ± 0.82	1.45 ± 0.68	1.48 ± 0.73	1.31 ± 0.54	1.35 ± 0.54	1.25 ± 0.54
The first three most frequently detected PIMs	1.PPI	1.PPI	1.Quetiapine	1.PPI	1.PPI	1.PPI	1.PPI	1.Doxazosin
	2.Quetiapine	2.Trimetazidine	2.Diltiazem	2.Quetiapine	2.Quetiapine	2.Quetiapine	2.Quetiapine	2.Nifedipine
	3.Trimetazidine	3.Diltiazem	3.Tamsulosin/doxazosin	3.Doxazosin	3.Doxazosin	3.Digoxin	3.Doxazosin	3.Haloperidol
T-value with dependent *T*-test between the proposed tool and criteria		12.8	12.2	11.47	18.23	9.62	14.5	13.6
*p-*value		<0.001						

It was calculated that a total of 7.3 ± 6.77 drug–drug interaction warnings should be given per patient with the proposed tool. As given in Table 3, the rate of patients with drug–drug interaction information of at least two of them was 89.2% (*n* = 265). According to the medication prospectuses which were included in the proposed tool database, the rate of patients with at least one contraindicated drug–drug interaction was 4.73% (*n* = 14). There are an average of 4.36 ± 3.82 warnings about drug-disease interaction per patient. The proportion of patients who have at least one interaction information with the chronic disease of one of the medicines they use is 87.8% (*n* = 260). According to the medication prospectuses which were included in the proposed tool database, the rate of patients with at least one contraindicated drug-disease interaction was found to be 11.8% (*n* = 35). The most common contraindicated drug-disease interaction is the use of quetiapine in the case of dementia. Drug–drug interaction information and drug-disease interaction information determined with the proposed tool are higher than the other three criteria, and this is statistically significant according to the dependent groups’ *t*-test (*p* < 0.05).

**Table 3:** Comparison of drug–drug and drug-disease interaction coverage according to each criterion and according to the proposed tool as the reference method.

	Reference Method	AGS BEERS 2019	TIME-TO-STOPP	STOPP V2
Average drug–drug interaction information per patient	7.3 ± 6.77	0.48 ± 0.25	0.03 ± 0.17	0.09 ± 0.3
Drug–drug interaction ratio (%)	89.2%	4.8%	4.03%	7,25%
Contraindicated drug–drug interaction ratio (%)	4.73%			
The t values with dependent *t*-test in determining drug–drug interaction information between the proposed tools and criteria		11.8	11.9	11.8
*p*-value		0.05	<0.05	<0.05
Average drug-disease interaction information per patient	4.36 ± 3.82	0.64 ± 1.07	0.065 ± 0.25	0.09 ± 0.4
Drug-disease interaction ratio (%)	87.8%	0.38%	7.25%	7.25%
Contraindicated drug-disease interaction ratio (%)	11.8%			
The t values with dependent *t*-test in determining drug-disease interaction information between the proposed tools and criteria		11.3	12.4	12.3
*p*-value		<0.05	<0.05	<0.05

Pearson and Spearmen correlation analyzes were performed on the number of PIM according to the reference method (regardless of the disease and other medicines used), patient age, number of chronic diseases, and number of medicines used by patients. When we look at the results of the correlation analysis in Table 4, no significant relationship was found between age and the number of chronic diseases (*p* = 0.347), between age and the number of medications used (*p* = 0.544), and between age and the amount of PIM detected by the proposed tool (*p* = 0.548). There is a significant positive correlation between chronic disease and the medication used, and this relationship is moderately strong (*p* < 0.001, r = 0.416). There is a significant positive correlation between chronic disease and the number of PIM use, and this relationship is weak between them (*p* < 0.001, r = 2.218). A significant positive correlation was also found between the number of medications used and the number of PIM use, and it can be said that this relationship is strong between them (*p* < 0.001, r = 0.605).

**Table 4:** Correlation analysis between the number of PIM according to the proposed tool as the reference method, patient age, the number of chronic diseases, and the number of medicines used according to the sample data.

	Correlation analysis									
	The number of PIM according to the proposed tool				The number of medicines				The number of chronic diseases	
	Pearson	*p*-value	Spearman	*p*-value	Pearson	*p*-value	Spearman	*p*-value	Pearson	*p*-value
Age^*^	−0.042	0.474	−0.035	0.548	−0.035	0.544			0.055	0.347
The number of chronic diseases^*^	0.218	<0.001	0.273	<0.001	0.411	<0.001	0.416	<0.001	1	
The number of medicine^*^	0.605	<0.001	0.646	<0.001	1					

* They show normal distribution according to the Skewness–Kurtosis test.

The correlation analysis results between the knowledge of drug–drug and drug-disease interaction detected determined by the proposed tool as a reference method and the chronic disease and the number of drugs is given in Table 5. According to the results, there is a significant positive correlation between the number of chronic diseases and drug-disease interactions, and there is a strong relationship between them (*p* < 0.001, r = 0.794).

**Table 5:** Correlation analysis between drug–drug and drug-disease interaction information determined according to the proposed tool, and the number of chronic diseases and medications according to the sample data.

	Correlation analysis			
	The number of chronic diseases		The number of medicines	
	Pearson	*p*-value	Pearson	*p*-value
Drug–drug interaction information according to the proposed tool	0.414	<0.001	0.794	<0.001
Drug-disease interaction information according to the proposed tool	0.362	<0.001	0.457	<0.001

A physician’s printout time to detect PIM (drug-age interaction), drug–drug interaction, and drug-disease interaction of each patient of 296 patient data using the recommended web application and a physician’s printout time for detecting interactions in three categories without the web application by manually scanning current articles, guidelines, and 6 PIM criteria [AGS Beers 2019, STOPP v2, Time-to-STOPP, EU(7)-PIM, Priscus, US-FORTA] in the web application database were compared. According to this, the printout time it took for a physician to detect PIM, drug–drug interaction, and drug-disease interaction for each patient by scanning the literature on the Google search engine without the web application was 3 min 13 s in minimum, 68 min 17 s in maximum, and on average 37 min 58 s (2,278 ± 856 s). On the other hand, the time it took to detect PIM, drug–drug interaction, and drug-disease interaction for each patient with this web application was found to be a minimum of 3 s, maximum of 65 s, and an average of 33.8 ± 15.8 s. Pearson correlation analysis results show the relationship between the number of chronic diseases, the number of medications, and printout time of interaction detection with and without the web application. Moreover, dependent group t-test analysis results to show the relationship between the time spent without and with the web application are given in Table 6. According to this table, there is a positive correlation and a strong relationship between the time it takes a doctor to detect interaction in a patient without or with the help of the proposed web application, and there is a positive correlation and a very strong relationship between the duration and the number of medicines. These relationships are statistically significant (*p* < 0.001). While the time it took was 2,278 s on average for a physician to detect interactions per patient without the web application, this time decreased to 33.8 s on average per patient with the proposed web application, and this is statistically significant (*p* < 0.001).

**Table 6:** Dependent groups t-test analysis and correlation analysis between the number of chronic diseases, the number of medications, and printout time of interaction detection with and without web application.

	Correlation analysis						Dependent groups *T*-Test analysis			
	The number of chronic diseases		The number of medicines		The total number of chronic diseases and medicines		Average	Std. deviation	*t*-test value	*p*-value
	Pearson	*p*-value	Pearson	*p*-value	Pearson	*p*-value				
Printout time without web application (s)	0.759	<0.001	0.916	<0.001	0.962	<0.001	2,278	856	24.1	<0.001
Printout time with the web application (s)	0.745	<0.001	0.830	<0.001	0.894	<0.001	33.8	15.8		

## Discussion

Advanced age and multimorbidity are predisposing factors in increasing polypharmacy and polypharmacy-related side effects (53). The possibility of ADEs due to PIM and drug–drug interactions is higher in the patient population with multimorbidity, advanced age, and polypharmacy (54). This situation also causes increased treatment costs, increased hospitalization, and increased mortality (1, 4, 7, 11, 12). Drug side effects increase with the number of medicines used; therefore, the presence of polypharmacy causes more undesirable ADEs and drug interactions (9). In a study conducted in 2008 to determine the risk of developing side effects with the number of medicines used, the risk of developing side effects is 15% with the use of two medicines, this rate is 58% with the use of five medicines, and it goes up to 82% with the use of seven or more medicines (55, 56). As one of the complications of polypharmacy, PIM use increases in the presence of polypharmacy, and the risk of ADEs also increases (13). In this study, the mean age of the sample was 65–103, the mean age for men was 83 ± 8, and 86 ± 7 for women. The polypharmacy rate of the patients was 64.9% (*n* = 192) and the multimorbidity rate was 97.3% (*n* = 288). The polypharmacy rate in this study was similar to previous elderly polypharmacy status determination studies (51, 57). As the number of chronic diseases of the patients and the number of medicines they use increase, the number of drug–drug interactions and drug-disease interactions also increase. A significant positive correlation was found between these data. In light of this information, it can be said that similar to the literature, chronic diseases and the amount of medication increase the side effects of polypharmacy. In this study, no significant relationship was found between age and the amount of medication, between age and the number of chronic diseases, and between age and the amount of detected PIM. This may be because the sample consisted of patients aged 65 and over and in this age group polypharmacy and multimorbidity were already intensely observed.

According to the 2014 data of the Turkey Pharmaceuticals and Medical Devices Agency (TITCK), Prescription Information System, Family Physicians’ most often prescribed diagnosis to patients over 65 is “essential hypertension” (58). The most common diseases after hypertension (30.7%) in the elderly are osteoarthritis (20.4%), heart failure (13.7%), diabetes mellitus (10.2%), coronary artery disease (9.8%), and osteoporosis (8.2%) (59). The most commonly prescribed medicines by family doctors are non-steroidal anti-inflammatory drugs (NSAIDs), acetylsalicylic acid (ASA), lansoprazole, pantoprazole, and metoprolol, respectively (58). In a study conducted with 493 elderly patients in Turkey in 2020, the most common diagnoses of patients were hypertension, generalized anxiety disorder, diabetes mellitus, Alzheimer’s, and atherosclerotic heart disease. Also, the most commonly used drugs were found to be acetylsalicylic acid, paracetamol, pantoprazole, and metoprolol (13, 57). Similar to the literature, the most common chronic disease in this sample was hypertension (69.5%). Hypertension is followed by chronic kidney disease (42.2%), dementia (33.1%), and diabetes mellitus (29%), respectively. The top 5 medicines most frequently used by the patients in the sample were ASA (52.4%), proton pump inhibitors (46.6%), hydrochlorothiazide (40.7%), metoprolol (30.7%), and quetiapine (27%), respectively. In a study conducted in Turkey, it was determined that the most frequently prescribed medicine by family physicians for elderly patients was systemic diclofenac, and 5 of the first 21 drugs were NSAIDs (58). When compared to the literature, the frequently used medicines were similar, and the rate of patients using NSAIDs in this sample group (9%) was found to be much less than in the literature. Prolonged use of NSAIDs for more than 3 months, while there is an alternative treatment, is not suitable for elderly patients. There is a very high risk of gastrointestinal (GI) bleeding, ulceration, or perforation, which can be fatal; and there are cardiovascular contraindications. Therefore, it is important to reduce the use of NSAIDs in elderly patients in terms of rational drug use. ASA usage was found to be high in this sample, similar to the literature. In the case of using ASA for primary prophylaxis, the potential benefit and harm should be decided by weighing it, and reviewing the indications of patients using ASA may provide rational drug use (58, 60). Likewise, the rate of patients using PPI was found to be high, similar to the literature. However, PPI use is not appropriate in elderly patients due to multiple drug use. Long-term high-dose PPI treatment is associated with an increased risk of C. difficile infection and hip fracture. PPIs can be used for less than 8 weeks if there is an appropriate indication (e.g., oral corticosteroids or chronic NSAID use). The use of a full therapeutic dose longer than 8–12 weeks is not appropriate in the treatment of uncomplicated peptic ulcers or erosive peptic esophagitis (18). Similar to the literature, it is observed that metoprolol usage is higher in elderly patients. In the absence of comorbidities such as heart failure or ischemic heart disease, it is not recommended to use beta-blockers as the first-line treatment for hypertension in the elderly (58, 60). It is observed that the quetiapine usage rate of the patients in this sample is high. Except for schizophrenia and bipolar disorder, quetiapine use is inappropriate in elderly patients because of the increased risk of stroke, heart failure, pneumonia infection, and death (15, 18).

Due to age-related changes in the pharmacokinetic and pharmacodynamic metabolism of medicines, the drug-induced sensitivity of elderly patients may increase or decrease after their introduction into the body. For this reason, the medical treatment process for elderly patients is more complex (54). Over the past three decades, criteria/lists containing medicines that may be risky to use in the elderly were published around the world (9, 14, 61). These criteria are usually established through expert decisions, published reviews, and consensus techniques (62). The first of the most widely used of these criteria is the AGS Beers criteria, published in America in 1991 and updated periodically (9). One of the disadvantages of Beers criteria is that some medicines on the criteria list are outdated and out of use, and there is not enough evidence for some medicines to be included in the list (9, 63, 64). However, nearly half of the medicines listed in these criteria are not available in Europe or Asia countries, so new country-specific guidelines and criteria had to be developed by other countries. As the Beers criteria were not sufficient for practical use, STOPP/START criteria were published in Ireland in 2010, and TIME criteria were published in Turkey in 2020 (17, 18, 65, 66). In addition to these, FORTA (Fit For The Aged), PRISCUS, EU (7)-PIM, NORGEP (The Norwegian General Practice), and Laroche criteria are accepted in various countries as potential inappropriate drug use criteria in elderly patients (19–24, 67).

In the literature, there are studies investigating PIM use in elderly patients and comparing PIM criteria with each other. As a result of a study conducted in 2006 in America with 193 patients over 65 on PIM use, 65% of them use one or more inappropriate drugs. In addition, 37% of patients used PIMs, and 57% of them use medicines that are ineffective, not indicated, or have a similar effect according to the Beers criteria (68). In a study in 2018 where 493 geriatric patients were evaluated according to the Beers and STOPP criteria, PIMs were detected in 34.4% of the patients (51). In a study where 493 geriatric patients were screened according to the Beers criteria in 2020, the most commonly used PIM was found to be quetiapine (13, 57). The rate of patients in sample data in this study using at least one or more PIM was found to be 75.3%, with the proposed tool that evaluated the 6 PIM criteria together. The most commonly used PIMS are PPIs, quetiapine, and trimetazidine, respectively. The rates of at least one or more PIM use by the patients in this sample were determined as (63.5%–19.5%) according to EU(7)-PIM, US-FORTA, Time-to-STOPP, AGS Beers 2019, STOPP v2, AGS Beers 2012, and Priscus criteria, respectively. These rates were concluded to be similar to the literature. It is seen that the coverage rate is higher with the proposed tool. This is due to the fact the proposed tool evaluates six criteria together and contains more information about the medicine’s use in elderly patients.

In a study conducted with geriatric patients in Turkey, the PIM coverage rate using STOPP criteria (39.1) was higher than the Beers 2012 criteria (33.3) (69). In another study, the rate of patients with PIM was found to be higher in Beers 2015 (49.5%) compared to STOPP (46.1%) criteria (51). In a study in which patients over 65 were screened according to the Beers 2015 and Beers 2019 criteria, and more PIMs were found according to the 2019 AGS Beers criteria (13, 57). When the literature is reviewed, the scope of Beers criteria in terms of containing PIM increased with each update and approached the STOPP criteria (51). Similar to the literature, according to this study’s results, when the sample was screened for PIM, the coverage rates of Beers 2019 (55.7%), STOPP (51%), and Beers 2012 (48.6%) were close to each other, and it can be said that Beers passed the STOPP criteria by a small margin in terms of PIM coverage with updates. With the Beers updates, additions and removals to the medicine list are made according to the ones in the American pharmaceutical market. Although this situation seems good for the United States, the removal of some medicines during updates reduces the scope of medicines in the use of these criteria in other countries. For example, ticlopidine was removed from the 2015 AGS Beers with the 2019 update since it is no longer available in the American pharmaceutical market, but it is still actively sold in the Turkish pharmaceutical market (15).

In a study screening palliative care patients’ medications according to Beers 2019 and Time-to-Stopp Criteria in 2021, the number of patients with PIM was found to be higher in TIME-to-STOPP criteria than in Beers 2019 criteria (70). Similar to the literature, according to this study, for the rate of patients with PIM, the TIME-to-STOPP criteria (57.4%) was found to be higher than in Beers 2019 criteria(55.7%). The fact that some of the drug agents included in the Beers Criteria are available in the American pharmaceutical market but not in Turkey may explain the higher rate compared to the TIME-to-STOP criteria (70).

Inappropriate drug information in the elderly was expanded with the EU(7)-PIM list prepared based on Priscus, Laroche, Mcleod, Finnish, Beers 2012, and Stopp/Start criteria (24). In a study in 2018 in which the drugs used by elderly patients were screened with different criteria, the detection rate of PIM with EU(7)-PIM (37.4%) was higher than the AGS Beers 2015 (26.4%) and Priscus (13.7%) criteria (71). In a study comparing the criteria in 2016, more PIMs were detected with the FORTA criteria than with the Priscus and STOPP criteria. In addition, there is a great difference between the PIMs determined by the FORTA, Priscus, and STOPP criteria (72). In a study comparing PIM criteria in Germany, PIM use was found in EU(7)-PIM the most, followed by FORTA and Priscus (73).

In this study, 88 pharmaceutical agents found as PIM with the proposed tool as the reference method including six criteria together were screened separately with other 6 PIM criteria, and how many of these pharmaceutical agents were covered by the criteria were expressed on the Venn diagram in Figure 6. According to this diagram, regardless of the disease and other medicines used, the criteria with the highest coverage rate of PIM are EU(7)-PIM (76.1%), US-FORTA (60.2%), TIME-to-STOPP (43.2%), AGS beers 2019 (41%) and PRISCUS (25%), respectively. Of the contained PIMs, 12% were identified within all six criteria. Similarly in the literature, in a study in which inappropriate drugs were found with 3 different criteria, the rate of PIMs that FORTA, Priscus, and EU(7)-PIM determined together constituted a small portion (6.7%) (73). This suggests that using a single PIM list for PIM detection is not sufficient and leads to the assumption that existing PIM lists should be expanded. Almost all (96%) of the PIMs contained with Priscus were identified by the EU(7)-PIM list. This situation was similar to the one in the literature (73). In this sample group, the PIM coverage rate determined by the EU(7)-PIM and US-FORTA criteria alone is 12% for each. As a result, similar to the literature, there is only a small similarity between all PIM lists in this study, and it can be said that there is wide heterogeneity in PIM detection in addition to some classical drugs. In addition, among the criteria, the ADE risk of the medicine in the elderly patient may vary according to the duration and dose of drugs. The criteria that do not specify the risk of medicine use depending on the dose and duration are not sufficient in practice. In addition, according to some criteria, this condition is considered inappropriate for the use of some drugs in elderly patients, and appropriate for others. For example, long-term use of NSAIDs for longer than 3 months, corticosteroids for 3 months, PPIs for 8 weeks, benzodiazepines for 4 weeks, and colchicine for 3 months are risky in the elderly patient according to some criteria. This situation increases patient complexity and makes rational drug use difficult for clinicians. For example, ASA can be used in an elderly patient, but the complication risk increases depending on the dose and duration. ASA may aggravate existing GI ulcers or produce new GI ulcers. There is an increased risk of bleeding due to prolonged clotting time, elevated INR values, or inhibition of platelet aggregation. Therefore, doses above 325 mg are in the potentially inappropriate drug category in the elderly (24). Chronic use of ASA at doses higher than 75–150 mg/day for primary or secondary cardiovascular protection should be avoided if other alternatives are not effective and the patient does not receive gastric protective treatment (15, 18, 74). Risperidone can be given as another example. The use of risperidone, an antipsychotic drug, for more than 6 weeks was defined as potentially inappropriate drug use in an elderly patient (15, 24). It can be given as a treatment for less than 6 weeks. It is recommended to use the lowest possible dose (0.5–1.5 mg/d) for the shortest possible time (24). It should be avoided except for schizophrenia or bipolar disorder, or for short-term use as an antiemetic during chemotherapy (15). There is an increased risk of mortality in dementia patients at higher doses because of the problematic risk–benefit profile for the treatment of behavioral symptoms of dementia (18). As a result, the risk or non-risk status of the drug in an elderly patient depends on the duration of use and dose of the medicine.

The content of the TIME-to-STOP/START and STOPP/START criteria is arranged not as a list of inappropriate drugs, unlike Beers, FORTA, Priscus, and EU(7)-PIM, but as a general recommendations list on medicine groups depending on the disease, duration, and dose. That makes it easier to make patient-centered decisions, but more difficult to use in clinical practice for a clinician. One reason for this is that the recommendations for the medicine group instead of the pharmaceutical agent may cause a more time-consuming process for the clinician. For example, the “NSAIDs are not suitable to be used for longer than 3 months when there is alternative treatment” recommendation in the TIME-to-STOPP criteria points to the NSAID medicine group but does not provide information about the pharmaceutical agent used by the patient. This situation complicates the applicability of the criteria to practical use.

As can be seen in the literature and in this study, the PIM criteria alone are insufficient to include actively used medications and they show heterogeneity (25). In addition, the inadequacy of criteria for PIM, drug–drug interaction, and drug-disease interaction causes difficulties in evaluating rational drug use in clinical practice. For this reason, there is a need for more comprehensive, patient-centered, system-based, and easily applicable tools containing information on medicine dose and duration information.

It is estimated that one out of six elderly patients is exposed to drug–drug interactions. There are many studies analyzing drug–drug interactions in elderly patients according to STOPP/START and Beers Criteria (54). In studies conducted in Europe, the rate of incorrect medicine combinations in elderly patients (9.8%–38.5%) was found to be relatively higher than the rate of incorrect medicine combinations in the United States (21.3%–28.8%) (54, 75–78). In a study conducted in primary care by evaluating the electronic records of 24,619 patients in Germany, the potential drug-disease interaction frequency was found to be 10.4% according to the Beers Criteria. Also, according to the results of this study, the drug-disease interaction rate increases in patients using 4 or more medicines (79). With the proposed tool, there is an average of 7.3 ± 6.77 (89.2%) drug–drug interaction warnings per patient in this sample group. In addition, the rate of patients using at least one dangerous medicine combination was found to be 4.73% (*n* = 14). Compared to the literature, the number of patients with drug interaction was found to be higher in this study, depending on whether the drug–drug interactions are mild, moderate, or dangerous. It can be said that the reason for this is that in addition to dangerous drug–drug interactions that cause the change or discontinuation of the drug, mild and moderate drug–drug interactions that require close clinical follow-up are also included in the proposed tool. According to AGS Beers 2019, TIME-to-STOPP and STOPP v2 criteria, the rates of patients with at least one drug–drug interaction information were found to be 4.8%, 4.03%, and 7.25%, respectively. The drug–drug interaction information included with the proposed tool is higher than the other 3 criteria, and this is statistically significant according to the dependent groups’ t-test. According to AGS Beers 2019, TIME-to-STOPP, and STOPP v2 Criteria, the rates of patients with at least one drug-disease interaction information were determined as 0.4%, 7.25%, and 7.2%, respectively. The drug–drug interaction information included with the proposed tool is higher than the other three criteria, and this is statistically significant according to the dependent groups’ *t*-test. In addition, the proposed tool includes all drug–drug and drug-disease interactions detected with AGS Beers 2019, TIME-to-STOPP, and STOPP v2 criteria. As a result, it can be said that the recommended tool is more comprehensive than the PIM criteria, which includes drug–drug and drug-disease interactions.

There are major side effects of polypharmacy including drug–drug and drug-disease interactions. For this, there are PIM criteria, medical books, medication package inserts, and the current guidelines for the treatment of chronic disease, as well as drug–drug interaction detection applications that can be used online (36–42). Examples of these drug interaction websites are UpToDate, Lexicomp, Vademecum online, Medscape online drug interaction, Webmd drug interaction, and DDInter. The comparison of these applications and the proposed web application *(Fast&Rational)* in this study are given in Table 7. As can be seen in the table, the existing auxiliary tools and web applications only provide information about the interaction of the medicines with each other used by the patient, rather than evaluating the patient holistically along with their diseases and age. In addition, some of the auxiliary tools in the literature are paid and some do not show any bibliography on interaction information. Turkish language support is also important for local users who do not have a sufficient level of English. These situations reduce the accessibility, ease of use, and reliability of these applications in practice. In Turkey, in 2012, the e-prescription system, which is a technology that electronically records all the format information of the prescription in a common system, was introduced (80). After the physician writes the patient’s prescription electronically on the e-prescription system, the system automatically warns about the drug–drug interaction in the prescription. This system is an important step forward in the name of rational drug use for this country. However, the warning system on the e-prescription system ignores the medicines that the patient uses regularly but were not included in the prescription at that time, and only gives information about drug–drug interaction. The proposed web application provides a more comprehensive approach, evaluates a geriatric patient’s age, medications and diseases together, and presents the user with advanced age-drug, drug–drug, and drug-disease interactions together. The fact that this application is a free web application and has Turkish and English language support makes it easily accessible and easy to use in practice. In addition, while presenting interaction information, it cites the resources and that increases the reliability. In the case of detection of interactions by the web application, offering alternative pharmaceutical agents according to patient’s age, medicines, and diseases by the proposed AI-based web application will be added in the next versions.

**Table 7:** The comparison of the proposed web application in this study and other web applications for detecting drug interactions.

	Drug-age interaction (PIM)	Drug–drug interaction	Drug-disease interaction	Provide resources	For free	Local language-supported	English language support	Offering alternative pharmaceutical agents
The proposed web app^*^	+	+	+	+	+	+	−/+**	−/+**
UpToDate Lexicomp	−	+	−	+	−	−	+	−
Vademecum online	−	+	+	−	−	+	−	−
Webmd	−	+	−	−	+	−	+	−
Medscape	−	+	−	−	+	−	+	−
DDInter	−	+	−	+	+	−	+	+

* The proposed web application is publicly available at https://fastrational.com/.

** These are the features that were not in the first version of the proposed web application but are planned to be added in the next versions.

Deprescribing refers to the process of ensuring the safe and effective use of medicines by a healthcare professional by gradually reducing inappropriate medicines to reduce polypharmacy and its side effects. This concept is an important part of rational drug use and therefore quaternary protection (28, 29). Deprescribing aims to reduce inappropriate drug use, the risk of falls, and the risk of hospitalization and death and aims to improve and/or maintain cognitive function (28, 81). This process is complex and requires attention, time, awareness, also special skills, and knowledge (28, 29). As a result of the systematic review of 40 studies between 2000 and 2019, the most important institutional barrier to “deprescribing” and rational drug use by family physicians is “the absence of evidence-based guidelines that clinicians can use in their practice in case of multiple diseases since evidence-based guidelines focus on single disease management” and “lack of assistive decision-making systems and tools” (82).

In the survey conducted in 2022 for family medicine residents in Turkey, in which the barriers to rational drug use in geriatric patients in their daily practice were questioned the participants stated, similar to the literature, that the most common barriers to rational drug use were “not knowing how to access resources,” although the resources on rational drug use were known beforehand, and “it is difficult and takes time to evaluate more than one source at the same time and make a patient-specific drug regulation” (83). In addition, in the study in which 75 clinicians in India were questioned about the PIM criteria in elderly patients, 90% of the participants stated that they did not know the criteria, but that these criteria could be effective in reducing clinically PIMs (84). As a result, it can be said that clinicians need auxiliary tools that will facilitate their access to up-to-date resources in daily practice, rather than taking their place in prescribing or drug regulation in an elderly patient. These auxiliary tools should be quick to apply, more comprehensive in terms of PIM, generalizable, and system-based, and should be also capable of evaluating patients’ current diseases and co-existing medical conditions together (25).

Today there are some online sites created with certain algorithms that will support and facilitate deprescribing elderly patients in the clinician’s practice (43–47). For example, deprescribing.org is an online application that offers prescribing algorithms for a limited number of medicine classes in Canada, but it can only be accessed within the system of contracted hospitals (45, 85). MedStopper is another deprescribing web application with an information system including Beers and Stopp Criteria. The user enters all pharmaceutical agents and indicates whether the patient is fragile or old. As a result, the application provides recommendations for pharmaceutical agents, symptoms that may develop in case of deprescribing, and dosage recommendations for the relevant agent in the Beers and Stopp criteria (44). Although it is an important tool for rational drug use, it provides more standard information rather than a patient-centered approach because it ignores the patient’s existing diseases. In this study, a tool is suggested that facilitates a more comprehensive, patient-centered approach and is easily accessible in clinical practice to support deprescribing.

In many studies in the literature, it was shown that the biggest obstacle to drug regulation in the case of polypharmacy is “time constraint” (83, 86, 87). A physician’s time to detect 6 PIM criteria (AGS Beers 2019, STOPP v2, Time-to-STOPP, EU(7)-PIM, Priscus, US-FORTA) on the database, PIM, drug–drug interaction, and drug-disease interaction of each patient with 296 patient data using current guidelines and medication package insert on the patient’s chronic diseases, using the proposed web application without application and with the web application were compared. Accordingly, without a web application, a clinician detects PIM, drug–drug interaction, and drug-disease interaction in a patient in approximately 2,278 s, with the web application this time decreased to an average of 33.8 s, which is statistically significant. It was aimed to facilitate the detection of patient-specific interactions and to provide quick access to the relevant bibliography with the proposed web application for the clinician.

One of the limitations of this study is that it is not known how long the patients were using the medicines in this sample. Herewith, it was accepted that the patients were using the pharmaceutical agents for at least 3 months when the data were analyzed since there may be variability between the criteria depending on the duration and dose of medicines.

In conclusion, the proposed web application in this study analyzes age, medication, and chronic diseases in approximately 34 s per patient and provides the opportunity to detect inappropriate drugs, drug–drug interactions, and drug-disease interactions approximately 100 times faster than a manual literature review. These evaluations show that the proposed web application will make great contributions to the solution of the time constraints problem in clinical practice.

The inadequacy and heterogeneity of criteria for PIM, drug–drug interaction, and drug-disease interaction causes difficulties in evaluating rational drug use in clinical practice. For this reason, there is a need for more comprehensive, patient-centered, system-based, and easily applicable tools containing information on medicine dose and duration information. According to the study results, it was shown that the proposed web application is 2 times more comprehensive than the most comprehensive criteria for inappropriate drugs in elderly patients and 5 times more comprehensive than the criteria with the lowest coverage in determining PIM.

PIMs, drug interactions, and drug side effects, which are among the side effects of polypharmacy, can cause limited health resources and the time of health workers to be wasted. The proposed web application, which is developed to reduce the side effects of polypharmacy and to facilitate rational drug use in practice, will bring along significant socioeconomic contributions with the improvements it will provide to the reduction of multimorbidity and mortality all over the world. There is a need for new studies to be carried out with the proposed web application and to calculate the returns at the world and national levels, and it is expected that this study and tool will be the subject of new studies.

Today, although there are important auxiliary tools and web applications for rational drug use, it provides more standard information rather than a patient-centered approach, since they ignore the patient’s existing diseases. With the proposed web application in this study, a more comprehensive tool is proposed that facilitates a patient-centered approach and is easily accessible in clinical practice, considering the patient’s age, medicines, and diseases. With this first and only artificial intelligence-supported web application, which is prepared with the 430 most used pharmaceutical agents in the market and outputs simultaneously in three categories, it is aimed to facilitate the detection of patient-centered interactions, provide quick access to the relevant bibliography, and ultimately to support the rational drug use for the clinicians.

## Data availability statement

The original contributions presented in the study are included in the article/supplementary material, further inquiries can be directed to the corresponding author.

## Ethics statement

The study (Document Date: 08/12/21, Document Number: E.Kurul-E2-21-1,083) was reviewed and approved by the Clinical Research Ethics Committee Presidency No.2 affiliated with Ankara City Hospital, Ankara Provincial Health Department, T.R. Turkey Ministry of Health.

## Author contributions

SA made the most contribution to the article, prepared the database of the web application, and made the statistic. The idea of the article belongs to SA and FA. FA designed and developed the AI-based web application and contributed to statistic. TY contributed to the discussion part and materials and methods part after SA and evaluated the article. All authors contributed to the article, reevaluated the article, and approved the submitted version.

## Conflict of interest

The authors declare that the research was conducted in the absence of any commercial or financial relationships that could be construed as a potential conflict of interest.

## Publisher’s note

All claims expressed in this article are solely those of the authors and do not necessarily represent those of their affiliated organizations, or those of the publisher, the editors and the reviewers. Any product that may be evaluated in this article, or claim that may be made by its manufacturer, is not guaranteed or endorsed by the publisher.
